# The WASH-complex subunit Strumpellin regulates integrin αIIbβ3 trafficking in murine platelets

**DOI:** 10.1038/s41598-023-36387-8

**Published:** 2023-06-12

**Authors:** Yvonne Schurr, Lucy Reil, Markus Spindler, Bernhard Nieswandt, Laura M. Machesky, Markus Bender

**Affiliations:** 1 Institute of Experimental Biomedicine-Chair I, University Hospital and Rudolf Virchow Center, Josef-Schneider-Str. 2, 97080 Würzburg, Germany; 2 Department of Biochemistry, University of Cambridge, Sanger Building, 80 Tennis Court Road, Cambridge, CB2 1GA UK

**Keywords:** Platelets, Integrins

## Abstract

The platelet specific integrin αIIbβ3 mediates platelet adhesion, aggregation and plays a central role in thrombosis and hemostasis. In resting platelets, αIIbβ3 is expressed on the membrane surface and in intracellular compartments. Upon activation, the number of surface-expressed αIIbβ3 is increased by the translocation of internal granule pools to the plasma membrane. The WASH complex is the major endosomal actin polymerization-promoting complex and has been implicated in the generation of actin networks involved in endocytic trafficking of integrins in other cell types. The role of the WASH complex and its subunit Strumpellin in platelet function is still unknown. Here, we report that Strumpellin-deficient murine platelets display an approximately 20% reduction in integrin αIIbβ3 surface expression. While exposure of the internal αIIbβ3 pool after platelet activation was unaffected, the uptake of the αIIbβ3 ligand fibrinogen was delayed. The number of platelet α-granules was slightly but significantly increased in Strumpellin-deficient platelets. Quantitative proteome analysis of isolated αIIbβ3-positive vesicular structures revealed an enrichment of protein markers, which are associated with the endoplasmic reticulum, Golgi complex and early endosomes in Strumpellin-deficient platelets. These results point to a so far unidentified role of the WASH complex subunit Strumpellin in integrin αIIbβ3 trafficking in murine platelets.

## Introduction

Platelets are small, anucleate and discoid-shaped cell fragments, which originate from mature bone marrow (BM) megakaryocytes (MKs). Platelets have a central role in hemostasis and thrombosis upon vessel wall injury^1^. Initial platelet adhesion is mediated via the interaction of the glycoprotein (GP) Ib-V–IX receptor complex present on platelets and collagen-immobilized von Willebrand factor (vWF). This only transient interaction allows other platelet receptors to bind to their ligands. Most importantly, it enables the interaction between GPVI and collagen, which triggers signaling pathways resulting in platelet activation and finally promotes a shift of β1 and β3 integrins from a low to a high affinity state for their ligands, allowing firm platelet adhesion and aggregation^2^. At least three major types of secretory granules (α-, δ-granules and lysosomes) are found in the cytoplasm of resting platelets, which differ in their ultrastructure, content, rate of exocytosis and function. α-Granules are highly abundant and contain, among others, coagulation factors, adhesive proteins, fibrinogen, vWF as well as αIIbβ3 and P-selectin, which are both expressed on the inner granule membrane^3,4^. Both δ-granules and lysosomes are low in number. δ-Granules contain, among others, calcium, ADP/ATP and serotonin, whereas lysosomes contain acid hydrolases^5^. Endo- and exocytic trafficking in MKs and platelets is crucial for proper platelet function as it enables cargo uptake and packaging into platelet granules, recycling of platelet surface receptors and the release of bioactive proteins upon platelet activation^6–8^. Furthermore, endocytic trafficking is important for platelet granule biogenesis and maturation, which occurs in MKs and platelets, respectively^8,9^. However, the mechanisms of endocytic trafficking, its molecular machinery and possible trafficking routes in MKs and platelets remain poorly understood.

The actin cytoskeleton is essential for many different cellular processes. Its formation and continual rearrangement are regulated by F-actin nucleating factors. Depending on their subcellular location, these nucleating factors induce local actin polymerization and thus, provide structural support, facilitate intracellular cargo trafficking and regulate membrane movement by generating mechanical force^10^. For instance, the actin cytoskeleton facilitates endocytosis at the plasma membrane as well as vesicle formation and transportation within the cell^11^. An important regulator of the actin cytoskeleton is the Arp2/3 complex, which has the ability to generate a network of branched actin filaments^12,13^. Lacking activity on its own, the Arp2/3 complex needs to be recruited and activated by nucleation promoting factors (NPFs)^13^. The ~ 500 kDa WASH complex (also known as the WASH regulatory complex; SHRC) is the major NPF on endosomes, where it regulates the formation of a branched actin network^10,14,15^. It consists of five subunits: WASH (also known as WASH complex subunit 1; WASHC1 or WASH1), FAM21 (family with sequence similarity 21; WASHC2, also known as KIAA0592), CCDC53 (WASHC3), SWIP (Strumpellin and WASH-interacting protein; WASHC4, also known as KIAA1033) and Strumpellin (WASHC5). The WASH complex associates with tubulin and localizes to early endosomal subdomains, which are enriched in Arp2/3 and F-actin^10^. WASH-generated F-actin not only regulates the architecture of the endolysosomal system, but also spares specific cargo from lysosomal degradation or missorting^16^. In *Drosophila* and mammalian cells it was shown that WASH1 has important functions in receptor recycling and lysosomal acidification^17,18^.

The WASH subunit Strumpellin is a 134 kDa protein, encoded by the *SPG8* (also known as *KIAA0196*) gene. In humans, several unique mutations in the *SPG8* gene have been associated with hereditary spastic paraplegia (HSP, also known as Strumpell-Lorrain disease), a group of inherited neurodegenerative diseases, whose main feature is a progressive gait disorder^19,20^. The loss of either WASH1 or Strumpellin in mice leads to embryonic lethality, emphasizing their function in development^21,22^. Recently, it was demonstrated that α5β1 integrin trafficking is altered in Strumpellin-depleted hTERT-RPE1 cells^23^. In general, however, the role of Strumpellin in cellular function is only poorly understood and its role in platelets is completely unknown.

Here, we generated MK- and platelet-specific Strumpellin-deficient mice and found a selective reduction of αIIbβ3 expression in MKs and platelets. αIIbβ3 was enriched in cellular compartments of Strumpellin-deficient platelets that share marker proteins with the endoplasmic reticulum, the Golgi apparatus and early endosomes, suggesting a disturbed endosomal machinery.

## Results

### Loss of Strumpellin results in decreased expression of integrin αIIbβ3 in platelets and MKs

**Figure 1:**
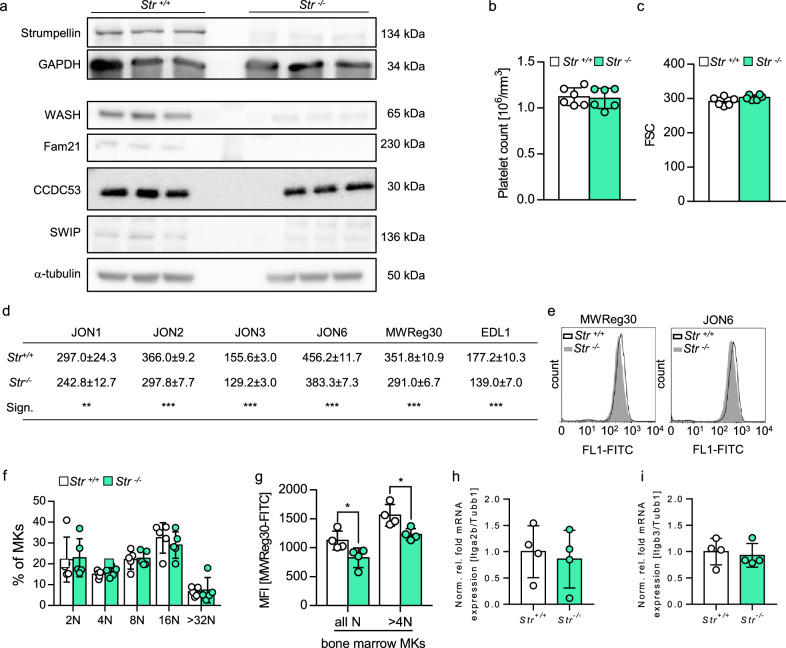
Loss of Strumpellin results in decreased expression of the integrin αIIbβ3 in platelets and megakaryocytes. (**a**) Immunoblot analysis of Strumpellin, WASH, Fam21, CCDC53 and SWIP protein content in platelet lysates from control (*Str*^+*/*+^) and Strumpellin-deficient (*Str*^*−/−*^) mice (n = 3). Glyceraldehyde 3-phosphate dehydrogenase (GAPDH) or α-tubulin served as loading control. (**b**) Platelet count and (**c**) size of peripheral blood platelets in control and Strumpellin-deficient mice measured by flow cytometry. Values are mean ± standard deviation (s.d.; n = 6). (**d**) Determination of the expression of αIIbβ3 on the surface of control and Strumpellin-deficient platelets with different antibodies recognizing the heterodimer αIIbβ3 (JON1, JON2, JON3, JON6 and MWReg30) or the β3 subunit (EDL1). Values are mean ± s.d. (n = 6; **P < 0.01;***P < 0.001). (**e**) Representative histogram of anti-αIIbβ3-FITC (MWReg30 and JON6) labeled platelet population from control (*Str*^+*/*+^) and Strumpellin-deficient (*Str*^*−/−*^) mice. (**f**) Ploidy level of primary BM MKs was assessed by flow cytometry. Values are mean ± s.d. (n = 5). (**g**) Surface expression of αIIbβ3 on primary BM MKs from all maturation stages (all N) and from MKs with ploidy greater than 4N (> 4N). Values are mean ± s.d. (n = 4; *P < 0.05). (**h, i**) Normalized fold mRNA expression of Itga2b (**h**) and Itgb3 (**i**) relative to MK specific mRNA Tubb1 from primary BM MKs based on qPCR. Values are mean ± s.d. (n = 4).

To investigate the role of Strumpellin in platelets, we generated MK- and platelet-specific Strumpellin-deficient mice (*Strumpellin*^*fl/fl, Pf4−Cre*^ further referred to as *Str*^*−/−*^; littermate controls *Strumpellin*^*fl/fl*^ further referred to as *Str*^+*/*+^) due to embryonic lethality of constitutive homozygous knockout mice^22^. The absence of the Strumpellin protein in mutant platelets was confirmed by Western blot analysis (Fig. 1a). We also used several antibodies against Strumpellin to study localization of the protein in platelets, which however, provided only no or unspecific staining. Next, we analyzed the expression of the other WASH complex members^24,25^. Immunostainings revealed a punctuated expression of WASH and CCDC53 in the platelet cytoplasm (Supplemental Fig. 1). Line profile analyses of confocal microscopic images of resting platelets indicate that the WASH protein is rather associated with α-granules (marker: vWF) and early endosomes (marker: EEA1) than with lysosomes (marker: Lamp1; Supplemental Fig. 2). In line with previous data in mammalian cells, in which RNA interference (RNAi)-mediated silencing of individual WASH complex subunits resulted in instability and degradation of other WASH complex components^10,14,24^, we found a decreased expression of WASH, CCDC53, FAM21 and SWIP in Strumpellin-deficient platelets by Western blot analysis (Fig. 1a). Count and size of platelets and other blood parameters were comparable between *Str*^+*/*+^ and *Str*^*−/−*^ mice as determined by flow cytometry (Fig. 1b,c) and a hematology analyzer (Supplemental Table 1). Next, surface expression of the major platelet integrin αIIbβ3 was determined by flow cytometry using specific FITC-labeled antibodies. Strumpellin-deficient platelets displayed a robust approximately 20% decrease in integrin αIIbβ3 surface expression, which was confirmed by different anti-αIIbβ3 antibodies (JON1, JON2, JON3, JON6, MWReg30) and one anti-β3 antibody (EDL1), excluding an antibody-dependent effect (Fig. 1d). Analysis of the histograms of αIIbβ3 surface expression (Fig. 1e) revealed that the entire population of the mutant platelets shifted towards a lower mean fluorescence intensity (MFI), suggesting a decreased αIIbβ3 integrin expression on every single Strumpellin-deficient platelet and not only on a platelet subpopulation. These results imply that Strumpellin regulates αIIbβ3 surface expression on mouse platelets. Of note, expression levels of the subunits of the integrin α2β1 are unaltered (Supplemental Fig. 3). Next, the mutant primary MKs were analyzed. The ploidy of *Str*^*−/−*^ MKs was comparable to controls indicating a normal MK maturation stage (Fig. 1f). In line with this, no significant changes in MK number were observed in bone marrow (Supplemental Fig. 4ai,ii) and spleen (Supplemental Fig. 4bi,ii) of Strumpellin knockout mice. However, we also measured an approximately 20% decrease of the MFI of αIIbβ3 on BM MKs by flow cytometry, indicating that the reduced αIIbβ3 integrin expression is passed on from MKs to platelets (Fig. 1g). Further, analysis of the mRNA levels of Itga2b and Itgb3 revealed no differences between Strumpellin-deficient and control MKs (Fig. 1h,i), excluding altered mRNA expression levels as a reason for the reduced surface expression of αIIbβ3. Taken together, Strumpellin regulates integrin αIIbβ3 expression in murine platelets and MKs.

**Figure 2:**
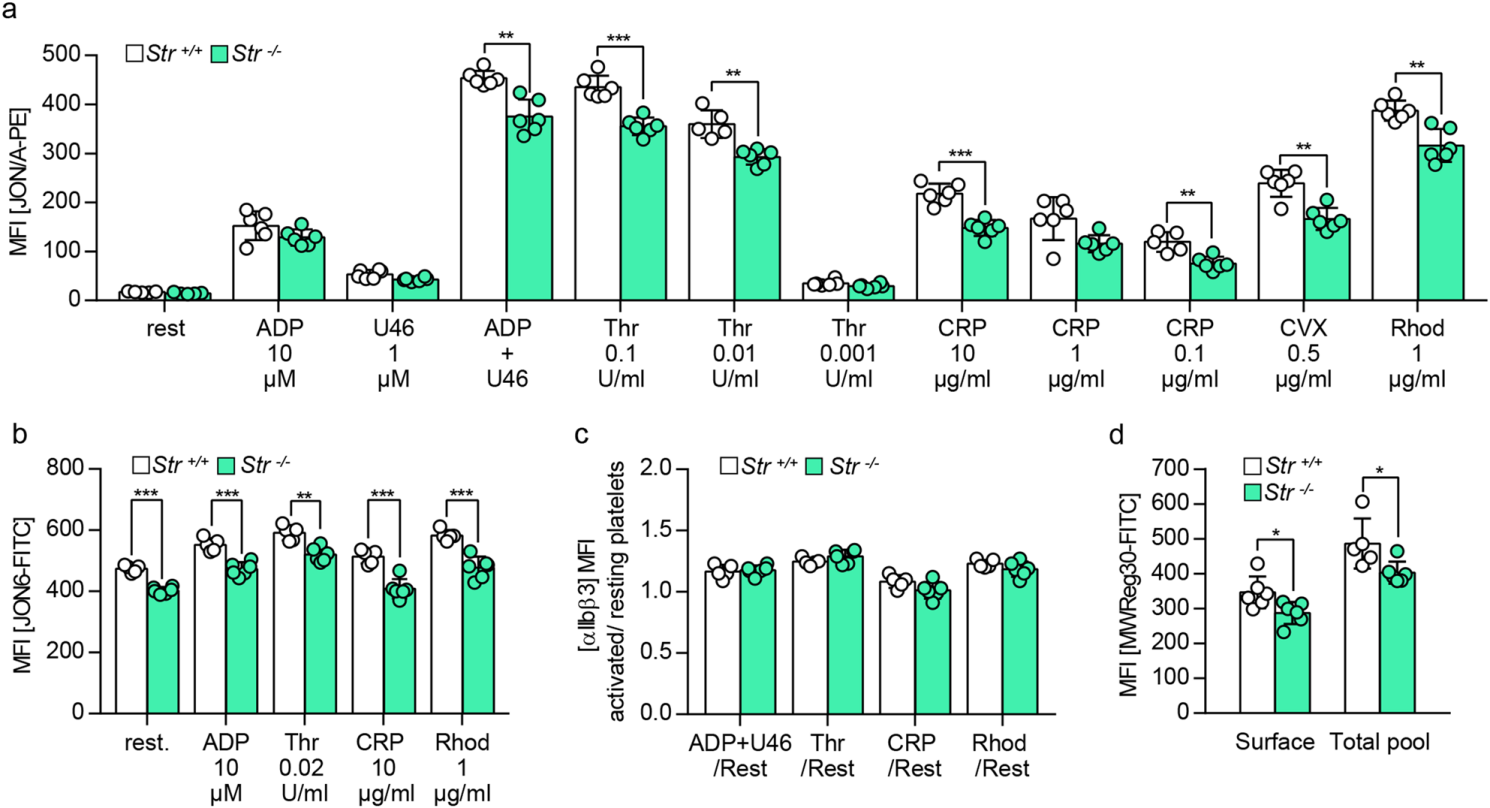
Moderately reduced αIIbβ3 activation but normal recruitment of internal αIIbβ3 pool to the plasma membrane after platelet stimulation with different agonists. (**a**) Determination of αIIbβ3 activation on the surface of resting platelets and upon stimulation with adenosine diphosphate (ADP), U46619 (U46, thromboxane analogue), thrombin (Thr), collagen related peptide (CRP), convulxin (CVX) and rhodocytin (Rhod) with specific antibodies (JON/A-PE) recognizing the active form of αIIbβ3. Values are mean ± s.d. (n = 6; **P < 0.01; ***P < 0.001). (**b**) Determination of total αIIbβ3 surface expression with specific antibodies (JON6) recognizing the heterodimer of αIIbβ3 under resting conditions and upon activation with adenosine diphosphate (ADP), thrombin (Thr), collagen related peptide (CRP) and rhodocytin (Rhod). Values are mean ± s.d. (n = 6; **P < 0.01; ***P < 0.001). (**c**) Ratio of total αIIbβ3 surface expression of activated versus resting platelets in (**b**). Values are mean ± s.d. (n = 6). (**d**) Surface expression and total pool of αIIbβ3 in platelets after permeabilization with antibodies (MWReg30) recognizing the heterodimer αIIbβ3. Values are mean ± s.d. (n = 6; *P < 0.05). Experiment in (**d**) was performed once.

**Figure 3:**
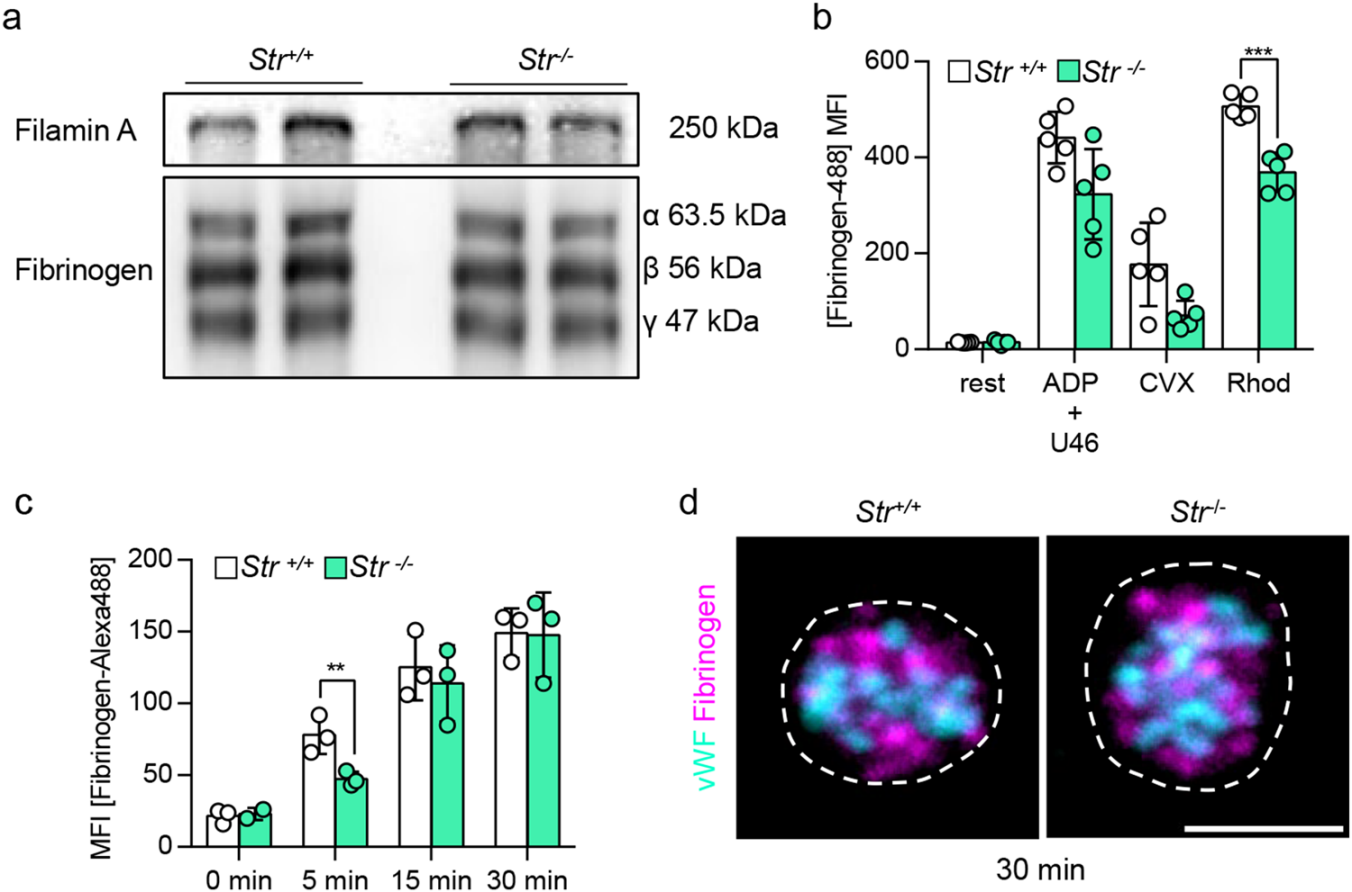
Normal fibrinogen content, but decreased fibrinogen binding upon activation and delayed fibrinogen uptake of mutant platelets. (**a**) Immunoblot analysis of fibrinogen content (α, β and γ-chain) of platelet lysates from two control (*Str*^+*/*+^) and two Strumpellin-deficient (*Str*^*−/−*^) mice. Filamin A served as loading control. (**b**) Binding of labeled fibrinogen (Fibrinogen-Alexa488) to platelets under resting conditions and upon activation with adenosine diphosphate (ADP) + U46619 (U46, thromboxane analogue), convulxin (CVX) and rhodocytin (Rhod). Values are mean ± s.d. (n = 5; ***P < 0.001). (**c**) Time dependent (0 min, 5 min, 15 min, 30 min) uptake of labeled fibrinogen by platelets. Values are mean ± s.d. (n = 3; **P < 0.01). (**d**) Representative confocal images of resting control and Strumpellin-deficient platelets, which were fixed after 30 min incubation with fluorescently-labeled fibrinogen (magenta). vWF staining (cyan) was used to visualize α-granules. Scale bar represents 1 µm.

**Figure 4:**
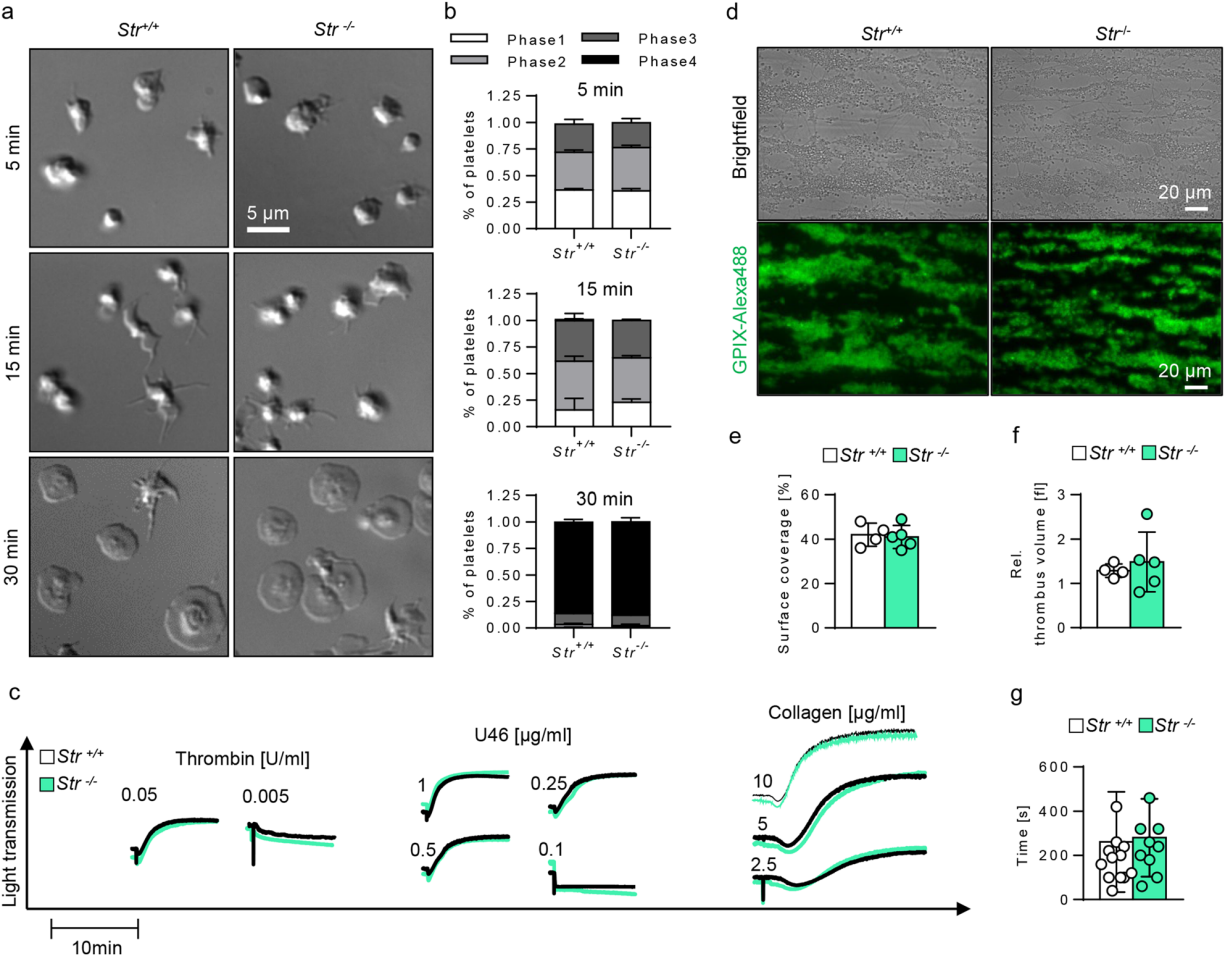
Normal in vitro platelet function after stimulation with specific agonists, ex vivo thrombus formation and in vivo hemostatic function. (**a**) Representative images of control and Strumpellin-deficient platelets spread on a fibrinogen-coated surface after 5, 15 and 30 min. (**b**) Percentage of platelets in the respective spreading phase (Phases 1–4) after 5, 15 and 30 min of spreading. Phase 1: round platelets; Phase 2: platelets with filopodia; Phase 3: platelets with filopodia and lamellipodia; Phase 4: platelets with lamellipodia. Values are mean ± s.d. (n = 3). (**c**) Representative aggregation curves of control and Strumpellin-deficient platelets after stimulation with different concentrations of thrombin, U46619 (U46, thromboxane analogue) and collagen. Values are mean ± s.d. (n = 4). This experiment was performed once. (**d**) Representative images after thrombus formation on a collagen-coated surface of control and Strumpellin-deficient platelets (shear rate 1000 s^−1^). Platelets were visualized with anti-GPIX-Alexa488 antibodies (lower panel). (**e**) Surface coverage and (**f**) relative thrombus volume of formed thrombi. (**e**,**f**) Values are mean ± s.d. (n ≥ 4). The experiments in (**d**–**f**) were performed once. (**g**) Bleeding time of control and Strumpellin-deficient mice after tail tip amputation. Values are mean ± s.d. (n ≥ 9).

### Recruitment of the internal αIIbβ3 pool after activation is normal in Strumpellin-deficient platelets

To evaluate if integrin αIIbβ3 activation is affected in the absence of the Strumpellin protein, we performed flow cytometry analysis of agonist-induced αIIbβ3 activation, which was slightly, but significantly reduced with almost all tested agonists (Fig. 2a), which might be due to the already reduced surface αIIbβ3 expression. After platelet activation internal αIIbβ3 receptors are translocated to the plasma membrane^26^. To examine, if the translocation of αIIbβ3 is altered in mutant platelets, αIIbβ3 expression was determined before and after platelet activation (Fig. 2b). Integrin αIIbβ3 surface expression was reduced on resting and activated *Str*^*−/−*^ platelets as tested with several specific anti-αIIbβ3 antibodies (Fig. 2b; Supplemental Fig. 5bi–gi), however, the expression ratio on activated/resting platelets was comparable to controls (Fig. 2c; Supplemental Fig. 5bii–gii). Of note, expression and ratio of β1-integrin on resting and activated mutant platelets were comparable to control platelets (Supplemental Fig. 5ai,ii). Moreover, permeabilization of resting platelets and subsequent staining for αIIbβ3 to measure the total αIIbβ3 pool also revealed an approximate 20% decrease of αIIbβ3 content (Fig. 2d). Our data implies that lack of Strumpellin leads to a reduced total pool of integrin αIIbβ3 in platelets, but αIIbβ3 recruitment to the platelet surface after activation is still functional.

**Figure 5:**
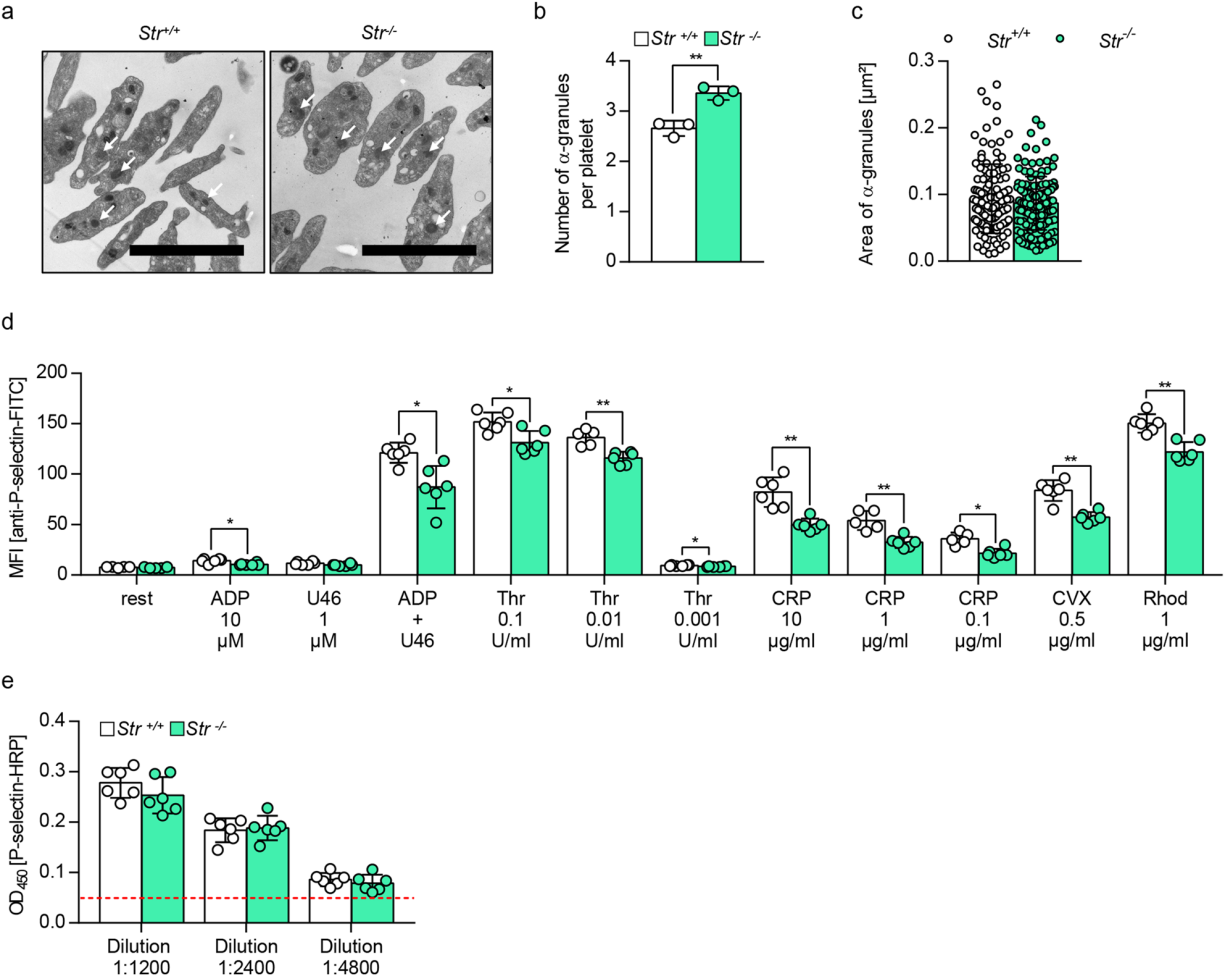
Reduced P-selectin exposure upon activation and increased number of α-granules in Strumpellin-deficient platelets. (**a**) Transmission electron micrographs from resting control and Strumpellin-deficient platelets. Exemplary white arrows point to α-granules. Scale bar indicates 3 µm. (**b**) Number of α-granules per platelet based on evaluation of transmission electron micrographs as shown in (**a**). Values are mean ± s.d.; in total at least 121 platelets from three control and three Strumpellin-deficient mice were analyzed (n = 3; **P < 0.01). The experiments in (**a**–**c**) were performed once. (**c**) Area of α-granules in platelets of control and Strumpellin-deficient mice based on transmission electron micrographs (**a**). Values are mean ± s.d. of at least 121 platelets (n = 3). (**d**) Determination of P-selectin exposure on the surface of resting platelets and upon stimulation with adenosine diphosphate (ADP), U46619 (U46, thromboxane analogue), thrombin (Thr), collagen related peptide (CRP), convulxin (CVX) and rhodocytin (Rhod). Values are mean ± s.d. (n = 6; *P < 0.05; **P < 0.01). (**e**) P-selectin content of resting control and Strumpellin-deficient platelets. Values are mean ± s.d. (n = 6). Dashed red line indicates the mean value from P-selectin-deficient platelets.

Next, we tested functional deficits of *Str*^*−/−*^ platelets due to the reduced integrin αIIbβ3 expression. Strumpellin-deficient platelets displayed a normal fibrinogen content (Fig. 3a), but fibrinogen binding to αIIbβ3 was moderately reduced (significantly for stimulation with rhodocytin, tendency for convulxin and ADP/U46619) (Fig. 3b). This is probably due to the decreased αIIbβ3 expression. Fibrinogen uptake into resting platelets was delayed within the first 5 min in *Str*^*−/−*^ platelets, but comparable to control platelets after 30 min of incubation with fibrinogen (Fig. 3c). In addition, we found a comparable distribution of endocytosed fibrinogen in control and mutant platelets (Fig. 3d). Furthermore, functional analyses of Strumpellin-deficient platelets revealed comparable spreading behavior in a static spreading assay on fibrinogen (Fig. 4a,b), aggregation in response to different agonists (Fig. 4c), as well as the capability to form ex vivo thrombi under flow on collagen (Fig. 4d–f) to control platelets. To examine the extent to which the reduced αIIbβ3 expression influences the hemostatic function, we performed a tail bleeding assay. No differences between *Str*^+*/*+^ and *Str*^*−/−*^ mice were observed (Fig. 4g), confirming previous data that heterozygous Glanzmann patients and heterozygous β3-integrin mutant mice, which only express 50% of wild-type αIIbβ3 in platelets, do not display a defect in hemostatic function^27,28^. These data indicate that αIIbβ3-dependent platelet functions are not affected in the absence of Strumpellin.

### Reduced P-selectin exposure after activation of Strumpellin-deficient platelets

Since several studies have reported that WASH1 or Strumpellin deficiency leads to an abnormal endo-lysosomal system^10,14,21,29^, we sought to investigate the αIIbβ3-containing α-granules of mutant mice in more detail. Analysis of transmission electron micrographs of platelets under resting conditions revealed an increased number of α-granules in Strumpellin-deficient platelets (Fig. 5a,b), however, area of α-granules was unaltered (Fig. 5c) and we could not observe obvious alterations in granule morphology (Fig. 5a). Next, we determined P-selectin surface exposure after platelet activation as a marker for platelet α-granule secretion. Platelets were activated with different GPCR, GPVI and CLEC-2-dependent agonists. *Str*^*−/−*^ platelets displayed marginally reduced P-selectin surface exposure after activation (Fig. 5d), whereas the total concentration of P-selectin in control and Strumpellin-deficient platelets was unaltered (Fig. 5e). This suggests a slightly impaired α-granule release in Strumpellin-deficient platelets.

### αIIbβ3 integrin is enriched in internal membrane compartments of Strumpellin-deficient platelets

**Figure 6:**
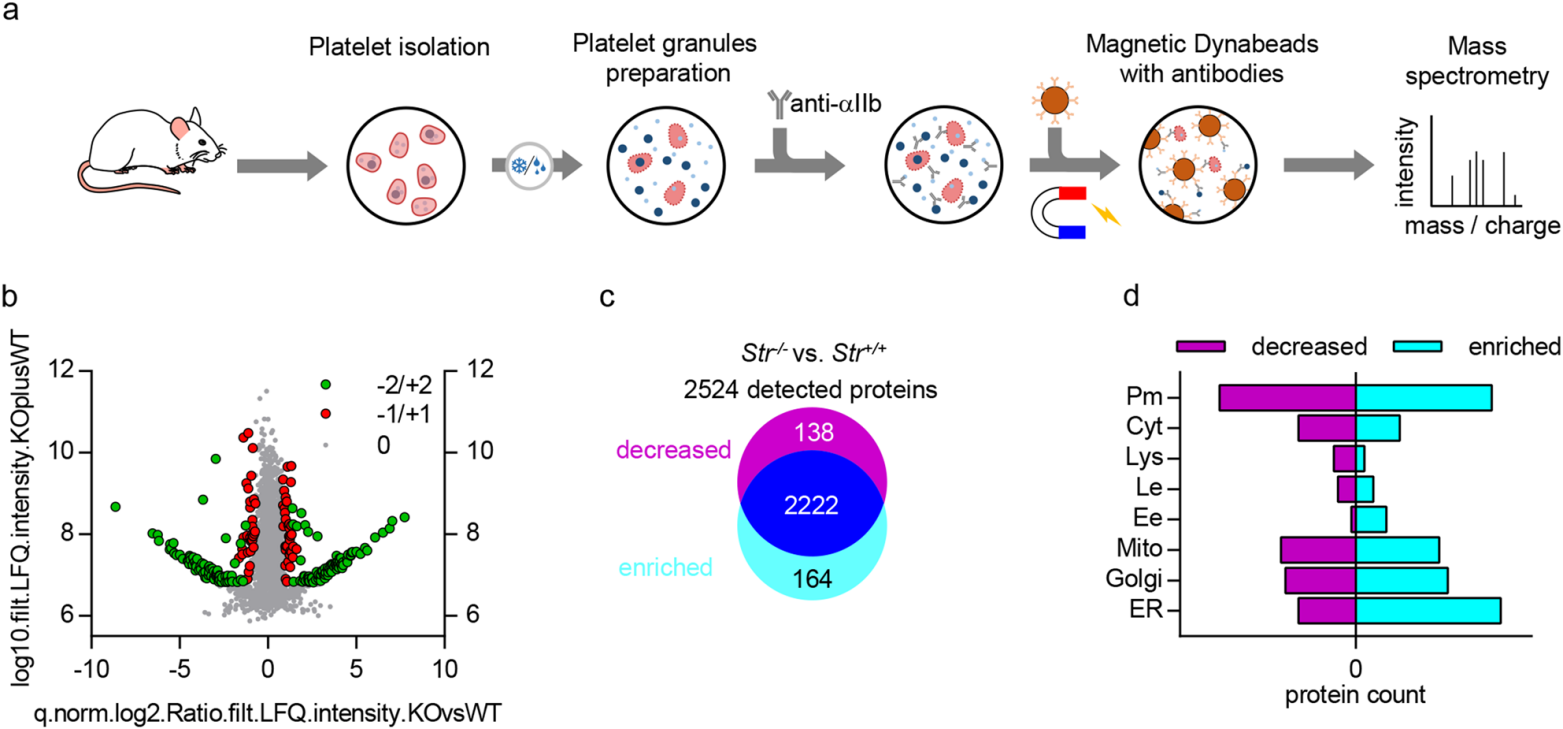
αIIbβ3 integrin is enriched in compartments of Strumpellin-deficient platelets that share marker proteins with early endosomes, Golgi apparatus and the endoplasmic reticulum. (**a**) Workflow of the platelet granules preparation and immunomagnetic sorting of αIIbβ3-positive granules with subsequent quantitative mass spectrometric analysis. (**b**) Quantitative mass spectrometry analysis of 2524 unique polypeptides from Strumpellin-deficient vs. control sample (for one sample platelets of 5 mice were pooled). Significant enrichment of unique polypeptides in the Strumpellin-deficient sample (green + 2 and red + 1 data points on the right side of the volcano blot) and significant reduction of unique polypeptides in the Strumpellin-deficient sample (green − 2 and red − 1 data points on the left side of the volcano blot) are depicted as well as polypeptides that were present in a similar quantity in both samples (0, grey data points). The experiment in (**b**) was performed once. (**c**) Distribution of the 2524 common polypeptides in control and Strumpellin-deficient samples. 138 unique polypeptides were reduced in the Strumpellin-deficient sample and 164 unique polypeptides were enriched in the Strumpellin-deficient sample. 2222 unique polypeptides were present in a similar quantity in both samples. (**d**) Databank analysis of the GOCC terms of the enriched and reduced unique polypeptides. From the 164 enriched polypeptides 33 were associated with the endoplasmic reticulum (ER), 21 with the Golgi apparatus, 19 with mitochondria (Mito), 7 with early endosomes (Ee), 4 with late endosomes (Le), 2 with lysosomes (Lys), 10 with the cytoskeleton (Cyt) and 31 with the plasma membrane (Pm). From the 138 decreased polypeptides 13 were associated with the endoplasmic reticulum, 16 with the Golgi apparatus, 17 with mitochondria, 1 with early endosomes, 4 with late endosomes, 5 with lysosomes, 13 with the cytoskeleton and 31 with the plasma membrane.

Our results thus far show that the total αIIbβ3 content in Strumpellin-deficient platelets is reduced by approximately 20% (Fig. 2d), however, transcription of ltga2b and ltgb3 is unaltered in MKs (Fig. 1h,i). We also detected full length proteins of αIIb and β3 of normal size and no shorter versions of these proteins in Strumpellin-deficient platelets, suggesting no alterations in αIIbβ3 maturation (Supplemental Fig. 6). Interestingly, the localization of early endosomes (EEA1), late endosomes (Rab7), slow recycling endosomes (Rab11) and lysosomes (Lamp1) was comparable between Strumpellin-deficient MKs and control samples (Supplemental Fig. 7). Next, we used immunomagnetic isolation of αIIbβ3-positive granules (containing α-granules, vesicles of the endosomes pathway, and membrane protein trafficking vesicles including ER, Golgi, and the plasma membrane) from a preparation of platelet granules and subsequently performed quantitative mass spectrometry (Fig. 6a). Quantitative comparison revealed that 138 unique polypeptides were decreased and 164 were increased in Strumpellin-deficient samples (Fig. 6b,c, Supplemental Excel file). Database analysis of the GOCC terms of the decreased and increased polypeptides showed that in Strumpellin-deficient platelets αIIbβ3 is enriched in compartments that share marker proteins with early endosomes, the Golgi apparatus and the endoplasmic reticulum (Fig. 6d). These proteomic data suggest that recycling of the integrin αIIbβ3 back to the plasma membrane is impaired and retrograde transport to the trans-Golgi network and endoplasmic reticulum is enhanced.

## Discussion

In this study, we describe for the first time the function of the Strumpellin protein in MKs and platelets. Our main conclusions are that (I) the WASH complex subunit Strumpellin is specifically involved in the regulation of αIIbβ3 receptor trafficking in MKs and platelets, while expression of other prominent receptors remains unaffected, (II) the integrin αIIbβ3 is enriched in compartments that share marker proteins with early endosomes, the Golgi apparatus and the endoplasmic reticulum in *Str*^*−/−*^ platelets and (III) platelet functionality is largely unaffected in the absence of Strumpellin.

We found a decreased expression of other WASH complex members in Strumpellin-deficient platelets pointing to an unstable WASH complex in *Str*^*−/−*^ platelets and most likely in MKs, too. Previous studies have shown that depletion of individual WASH complex subunits impacts overall stability of the complex. Nevertheless, some controversy exists concerning the destabilization of the entire complex or only of specific subunits as well as the residual activity. Data obtained from different cell lines suggested that subunits FAM21, SWIP and Strumpellin serve as core subunits upon which WASH1 and CCDC53 assemble^24^. However, complete knockout of WASH1 in mouse embryonic fibroblasts caused a significant reduction in protein levels of all WASH complex subunits^21^. Similar results were obtained in WASH1-deficient CD4-positive T-cells and hepatocytes^30^. Interestingly, residual FAM21, SWIP and Strumpellin still remained linked to each other in WASH1-deficient mouse embryonic fibroblasts, also pointing towards the assembly of a WASH core complex^21^. Furthermore, studies proposed that the remaining subunits still assemble and function, at least partially, after knockout of individual WASH complex subunits^29^. Altogether, these results suggest that depletion of WASH complex subunits leads to an unstable WASH complex due to partial degradation of remaining subunits, which however, can still show residual activity. It has been shown that Strumpellin also associates with spartin, a regulator of endosomal trafficking, as well as with valosin-containing protein (VCP) independently of the WASH complex^31,32^, suggesting WASH complex-independent functions of individual WASH components. Both proteins, spartin and VCP, are expressed in mouse and human platelets according to proteomics data^33,34^, however, their function in platelets is unknown. It cannot be excluded that WASH complex-independent roles of Strumpellin cause the reduced αIIbβ3 expression in mutant platelets. However, due to different studies reporting on the role of the WASH complex in integrin trafficking, it is more likely that Strumpellin regulates αIIbβ3 expression in a WASH complex-dependent manner.

Endocytic trafficking is an important regulatory mechanism to control integrin expression on the cell surface. Integrin αIIbβ3 is internalized into platelets and then recycled back to the plasma membrane for reuse^35^. Approximately 80,000 copies of αIIbβ3 are located on the platelet surface in resting platelets with an additional internal pool, which are always kept in a state of equilibrium^6^. Over the years, numerous platelet proteins have been identified that may contribute to endocytosis and/or subsequent cargo-sorting events (for detailed information, review of Banerjee et al.^6^). We identified Strumpellin as a new protein involved in the platelet endocytic machinery. Our flow cytometry analyses revealed a specific decrease of integrin αIIbβ3 surface expression on resting *Str*^*−/−*^ platelets and MKs, whereas the expression of other receptors was not affected. In vitro platelet spreading on fibrinogen or platelet aggregation were unaltered despite the 20% decrease in integrin αIIbβ3 expression. Moreover, bleeding times in a tail bleeding assay were comparable between control and the mutant mouse line demonstrating that the remaining 80% of αIIbβ3 are functional and sufficient for a normal hemostatic function. This is in agreement with results obtained from heterozygous β3-integrin mutant mice, which only express 50% of wild-type αIIbβ3 in platelets. These mice did not show any hemostatic defects, or alterations in platelet fibrinogen content and platelet aggregation^28^. Similarly, deletion of Arf6, an important regulator of αIIbβ3 trafficking, in mouse platelets did not affect hemostasis^7^. Moreover, hemorrhagic symptoms occur only in Glanzmann thrombasthenia patients with homozygous mutations^27^.

Why is there a decrease of only 20% of αIIbβ3 in MKs and platelets? One explanation could be that WASP family members substitute for one another. Like WASH1, WHAMM and JMY localize to internal membranes, where they are involved in maintaining organelle morphology and cargo transportation^36,37^. Furthermore, N-WASP is not only important for endocytosis, but also for endocytic vesicle and endosome movement through the cytoplasm^38,39^. Due to their functionally similar roles in intracellular transportation, it might be possible that WHAMM, JMY or WASP/N-WASP are able to compensate for the loss of WASH1 in Strumpellin-deficient platelets by upregulation and/or re-localization. On the other hand our data demonstrate that Strumpellin deficiency leads to a decrease, but not a loss of WASH1 protein in platelets. A more likely explanation is that in the absence of Strumpellin residual WASH complex subunits still assemble and partially function in αIIbβ3 trafficking. However, further experiments are needed to confirm either one or the other hypothesis.

We detected an enrichment of αIIbβ3 positive compartments in Strumpellin-deficient platelets that share marker proteins with endoplasmic reticulum, Golgi and early endosomes. Interestingly, we did not detect an accumulation of αIIbβ3 in lysosomes but rather a reduced number of polypeptides associated with lysosomes. One explanation might be that lysosomal activity is increased in Strumpellin-deficient platelets, resulting in a higher degradation rate of αIIbβ3 in platelets and therefore a decreased association of αIIbβ3 with lysosomes in the immunomagnetic sorting experiment.

In summary, we describe here for the first time the role of the WASH-complex subunit Strumpellin in platelets. We show that Strumpellin regulates integrin αIIbβ3 trafficking in murine platelets without affecting platelet spreading, platelet aggregation and hemostatic function.

## Methods

### Animals

All animal studies were approved by the district government of Lower Franconia (license number: 2-274). Methods were performed in accordance with the relevant guidelines and regulations. We followed the guidelines of ARRIVE (Animal Research: Reporting of In Vivo Experiments).

### Generation of ***Strumpellin***^***flox/flox***^ mice

Generation of *Strumpellin*^*flox/flox*^ was described by Tyrrell et al.^29^. To study the function of Strumpellin in platelets, *Strumpellin*^*fl/fl*^ mice were intercrossed with mice carrying the Cre-recombinase under the platelet factor 4 (*Pf4*) promoter^40^ to generate platelet- and megakaryocyte-specific Strumpellin-knockout mice. *Strumpellin*^*fl/fl Pf4−Cre*^ is further referred to as *Str*^*−/−*^, *Strumpellin*^*fl/fl*^ is further referred to as *Str*^+*/*+^ which served as controls.

### Preparation of paraffin sections and hematoxylin/ eosin staining

Five-micrometer-thick sections of formalin-fixed paraffin-embedded spleens and decalcified bones of male and female mice were prepared, deparaffinized and stained with hematoxylin and eosin. The number of MKs was analyzed with an inverted Leica DMI 4000 B microscope^41^.

### Platelet preparation

Mouse blood was collected in a reaction tube containing 20 U/ml heparin in TBS, pH 7.3^42^. Platelet-rich plasma (PRP) was obtained by two consecutive centrifugation at 80*g* for 6 min at room temperature (RT). PRP was supplemented with prostaglandin (PGI_2_; final concentration 0.5 µM) and apyrase (final concentration 0.02 U/ml) and centrifuged at 640*g* for 5 min. Platelet pellet was resuspended in 1 ml Tyrode’s buffer (134 mM NaCl, 0.34 mM Na_2_HPO_4_, 2.9 mM KCl, 12 mM NaHCO_3_, 5 mM HEPES, 1 mM MgCl_2_, 5 mM glucose, and 0.35% bovine serum albumin [BSA; pH 7.4]) containing 0.5 µM PGI_2_ and 0.02 U/ml apyrase and was centrifuged twice at 640* g* for 5 min. After adjustment of the desired platelet count, washed platelets with apyrase (final concentration 0.02 U/ml) were incubated for 30 min at 37 °C before they were used for experiments.

### Immunoblotting

Proteins of lysed platelets were separated by sodium dodecyl sulfate–polyacrylamide gel electrophoresis and blotted onto polyvinylidene difluoride membranes. After blocking, membranes were incubated with primary antibodies overnight at 4 °C (Supplemental Table 2). Horseradish peroxidase-conjugated secondary antibodies (Supplemental Table 2) and enhanced chemiluminescence solution (MoBiTec) were used for visualization^43^.

### Flow cytometry

#### Glycoprotein expression

Heparinized whole blood was diluted 1:20 in Tyrode’s-HEPES buffer, incubated with saturating amounts of fluorophore-conjugated antibodies for 15 min at RT and analyzed on a FACSCalibur (BD Biosciences, Heidelberg, Germany). A list of antibodies can be found in Supplemental Table 2. To determine platelet count and size, platelet populations of double positive cells (αIIbβ3 and GPV) were compared^43^.

#### Platelet activation

Blood samples were washed twice with Tyrode-HEPES buffer, incubated with agonists for 15 min in the presence of specific antibodies for active αIIbβ3 (JON/A, PE-conjugated, Emfret Analytics, Germany) and P-selectin (Wug.E9, FITC-conjugated, Emfret Analytics, Germany) for 15 min at RT, and analyzed on a FACSCalibur^43^. To determine the total pool of αIIbβ3, platelets were fixed and permeabilized with 2% paraformaldehyde (PFA) and 0.1% NP-40 before staining (MWReg30, FITC-conjugated, Emfret Analytics, Germany).

### Megakaryocyte ploidy

For determination of BM-MK ploidy levels, mice were sacrificed and femoral BM was flushed out in 2 ml CATCH buffer (25 mM HEPES, 3 mM EDTA, 3.5% BSA in PBS). Single cell suspension was obtained by pipetting the BM suspension up and down using a syringe and needles with different gauge (18G, 22G, finally 26G). The suspension was passed through a cell strainer (100 µm) to remove bone and other solid tissue parts. 200 µl of this suspension were used to obtain one sample per condition. Samples were centrifuged at 1200 rpm for 5 min at RT and the cell pellet was resuspended in 400 µl 1:1 mixture CATCH/PBS + 5% FCS. All samples were incubated for 15 min on ice with anti-CD16/CD32 antibodies (0.02 µg/µl; clone 2.4G2). All samples except control samples (pooled samples from each genotype) were incubated for 20 min on ice with FITC-labeled anti-αIIbβ3 antibodies (MWReg30) to stain the MK population. After adding 1 ml 1:1 mixture CATCH/PBS + 5% FCS per sample and centrifugation at 1200 rpm for 5 min at RT, the cell pellet was resuspended in 250 µl PBS with 0.1% EDTA and fixed with 250 µl PBS/1% PFA per sample for 10 min on ice. To remove the fixative, cell suspension was washed with 3 ml PBS per sample. After centrifugation at 1200 rpm for 10 min the cell pellet was resuspended in 500 µl PBS/0.1% Tween for 10 min on ice to permeabilize the cells. The washing step with 3 ml PBS was repeated. DNA was stained using propidium iodide (PI) (final concentration: 25 µg/ml) in H_2_O supplemented with RNase (final concentration: 100 µg/ml) over night at 4 °C in the dark. Analysis was performed on a FACSCalibur (BD Bioscience)^41^.

### MK cultivation and immunofluorescence of MKs and platelets

MKs/platelets were fixed, permeabilized and probed with primary antibodies against WASH protein (Invitrogen), EEA1, Rab7, Rab11 (Cell signaling), Lamp1 (Abcam), vWF (Dako) and Alexa488 or Cy3-conjugated secondary antibodies (Immuno JacksonResearch) to visualize the target proteins. DAPI and phalloidin were used to visualize the nucleus and F-actin cytoskeleton. The cells were imaged using confocal laser scanning microscopy (TCS SP8, Leica Microsystems CMS) with a 63×/NA 1.4 oil objective (MKs) and a 100×/NA1.4 oil objective (platelets). The LAS X software was used for image documentation and image processing was performed with Image J (Version 2.0.0-rc-43/1.51 g, NIH, MD, USA) software. Brightness and contrast adjustments were linearly applied to the whole image.

### Enrichment of BM MKs and qPCR

BM of both femora and tibiae of one mouse were collected by centrifugation^44^ and the primary mature BM MKs enriched per size exclusion using different cell strainers as follows: BM single cell suspension in BSA and FCS containing CATCH buffer were passed twice through a 20 µm cell strainer and remaining MKs in the filter were flushed out. The MK containing fraction was then filtered through a 15 µm cell strainer and collected. Subsequently, MKs were lysed and RNA was isolated with Trizol and the RNeasy Mini Kit from QIAGEN according to the manufacturer´s instructions. qPCR was performed with Syber Green from BIORAD using specific primers for ITGA2B (5′-GAGGCTGCTTCTTGGCTCAG-3′ 5′-AGCCTGCTTCACAGTATCGC-3′) and ITGB3 (5′-ACGCCATCATGCAGGCTAC-3′ 5′-CCAATGTGACAGTGCCCATC-3′). The expression was set in relation to MK-specific TUBB1 (5′-TAAGAAGTATGTGCCGCGAGC-3′ 5′-CATTTTCGATCAGCTCCGCTC-3′) expression and normalized.

### Fibrinogen binding and uptake assay

#### Fibrinogen binding

Washed platelets (18,000 platelets/μl) in Tyrode’s buffer containing 2 mM CaCl_2_ were either kept resting or were activated with a mixture of adenosine diphosphate (ADP) and the thromboxane analogue U46619 (10 μM/1 μM), convulxin (CVX, 0.5 μg/ml) or rhodocytin (Rhod, 1 μg/ml). Samples were then incubated with AlexaFluor 488-conjugated fibrinogen (Sigma, 50 μg/ml) for 5 min at 37 °C and finally 500 μl PBS buffer were added. Fibrinogen binding capacity of resting and activated platelets after the indicated time point was measured with a FACSCalibur.

#### Fibrinogen uptake

Washed platelets were centrifuged (5 min, 2800 rpm) and resuspended in Tyrode’s buffer (18,000 platelets/μl). To analyze the uptake of fibrinogen, as previously described^7^, platelets were incubated with AlexaFluor 488-conjugated fibrinogen (150 μg/ml) for 0, 5, 15 or 30 min at 37 °C. After fixation with 2% PFA overnight at 4 °C, 0.1% trypan blue was added to quench the extracellular signal of AlexaFluor 488-conjugated fibrinogen. Samples were incubated for 10 min before adding 500 μl PBS buffer. Intracellular AlexaFluor 488-fibrinogen levels were measured using a FACSCalibur. For qualitative analysis of fibrinogen uptake washed platelets were incubated with AlexaFluor 546-conjugated fibrinogen (100 µg/ml) for 30 min and then fixed/permeabilized with 1% PFA supplemented with 0.1% NP-40. Primary antibodies against vWF (Dako) and secondary antibodies (Immuno JacksonResearch) were used to visualize α-granules. Platelets were imaged using confocal laser scanning microscopy (TCS SP8, Leica Microsystems CMS) with a 100×/NA 1.4 oil objective. The LAS X software was used for image documentation and image processing was performed with Image J (Version 2.0.0-rc-43/1.51 g, NIH, MD, USA) software. Brightness and contrast adjustments were linearly applied to the whole image.

### P-selectin ELISA

A commercial mouse P-selectin enzyme linked immunosorbent assay (ELISA) kit was purchased from RayBio. The experiment was performed according to the manufacturer´s protocol to determine P-selectin content of platelet lysates. An antibody specific to murine P-selectin, coated on a 96-well plate, was used in this assay. If present in the sample, immobilized P-selectin was detected using a biotinylated anti-mouse P-selectin antibody, HRP-labelled streptavidin and TMB substrate solution. Platelet lysates from P-selectin-deficient mice were used as negative controls.

### Transmission electron microscopy

Washed platelets were fixed with 2.5% glutaraldehyde in 50 mM cacodylate buffer (pH 7.2). After embedding in epon 812, ultra-thin sections were generated and stained with 2% uranyl acetate and lead citrate. Samples were visualized with a Zeiss EM900 microscope^45^.

### Platelet spreading

For spreading on fibrinogen, coverslips were coated with 100 µg/ml human fibrinogen (Sigma) overnight at 4 °C and blocked with 1% BSA in PBS at 37 °C for 1 h. Slides were rinsed with Tyrode’s buffer before washed platelets were activated with 0.01 U/ml thrombin (Roche) and allowed to spread on the coated coverslips. Platelets were fixed with 4% PFA in PBS at different time points. Spread platelets were visualized with a Zeiss Axiovert 200 inverted microscope (100×/1.4 oil objective)^43^. Digital images were recorded using a CoolSNAP-EZ camera (Visitron) and analyzed using ImageJ software.

### Aggregometry

Washed platelets (160 µl with 0.5 × 10^6^ platelets/µl) were analyzed in the presence (U46619, collagen) or absence (thrombin) of 70 µg/ml human fibrinogen (Sigma). Light transmission was recorded on a four-channel aggregometer (Fibrintimer, APACT, Hamburg, Germany) for 10 min and expressed in arbitrary units^46^.

### Adhesion under flow conditions

Coverslips were coated with 200 µg/ml Horm collagen at 37 °C overnight, washed with PBS and blocked with 1% BSA in PBS for 1 h at 37 °C. Blood was collected in heparin (20 U/ml), diluted (2:1) in Tyrode’s buffer supplemented with Ca^2+^, and incubated with Dylight-488-conjugated anti-GPIX derivative (0.2 µg/ml) at 37 °C for 5 min. Transparent flow chambers with a slit depth of 50 µm, equipped with the coated coverslips, were connected to the blood-filled syringe. Perfusion was performed at shear stress equivalent to a wall shear rate of 1000 s^−1^. Blood was perfused for 4 min over the collagen-coated surface and washed with Tyrode’s buffer for 4 min^45^. Image analysis was performed using ImageJ software.

### Bleeding time

A 2 mm segment of the tail tip of an anesthetized mouse was removed with a scalpel. Tail bleeding was monitored by gently absorbing blood with a filter paper in 20 s intervals without making contact with the wound site. Once no more blood was observed on the paper, it was determined that bleeding had ceased. Otherwise, experiments were stopped after 20 min^43^.

### Immunomagnetic sorting of αIIbβ3 positive granules

Washed platelets from five mice per genotype were pooled and platelet granules isolated via alternating freezing and thawing cycles according to Nießen et al.^47^. Briefly, αIIbβ3 positive granules/vesicles and membranes from the protein trafficking machinery including ER, Golgi and the plasma membrane were isolated using specific antibodies against the cytoplasmatic tail of αIIbβ3 (Merck, Darmstadt, Germany) and magnetic dynabeads (sheep anti-rabbit, Thermo Scientific, Dreieich, Germany). Pulled down of granules/vesicles were eluated from dynabeads with 4% (w/V) SDS and associated polypeptides were analyzed by quantitative mass spectrometry.

### Data analysis

Data presented are mean ± standard deviation (s.d.) from at least two independent experiments per group or otherwise mentioned in the figure legend. Differences between control and knockout mice were statistically analyzed using the Mann–Whitney-U test. Asterisks indicate statistically significant differences compared with control (*P ≤ 0.05; **P ≤ 0.01; ***P ≤ 0.001). Results with a *P* value > 0.05 were considered as not significant.

Supplementary data are provided in the supplementary information.

## Supplementary Information

Supplementary Information 1.

Supplementary Information 2.

## Data Availability

The data generated and analyzed in this study are available from the corresponding author on reasonable request.
