# Supplementary material for: The WASH-complex subunit Strumpellin regulates integrin αIIbβ3 trafficking in murine platelets
Source: Sci Rep. 2023 Jun 12;13:9526. doi: 10.1038/s41598-023-36387-8 (PMC10260982; doi:10.1038/s41598-023-36387-8)
Supplement: Supplementary file 1 — Supplementary Information 1. [file 41598_2023_36387_MOESM1_ESM.pdf]

SUPPLEMENTARY INFORMATION

The WASH-complex subunit Strumpellin regulates integrin αIIbβ3 trafficking in murine platelets

Yvonne Schurr[1#], Lucy Reil[1#], Markus Spindler[1#], Bernhard Nieswandt[1], Laura M. Machesky[2] and Markus Bender[1*]

[1]Institute of Experimental Biomedicine – Chair I, University Hospital and Rudolf Virchow Center, Würzburg, Germany

[2]Department of Biochemistry, University of Cambridge, Sanger Building, Tennis Court Road, Cambridge, UK

[#]equal contribution

*Correspondence to: Markus Bender, PhD; Institute of Experimental Biomedicine, Chair I, University Hospital Würzburg; Josef-Schneider-Str. 2, 97080 Würzburg, Germany;
Phone: +49 931-201-48328; E-mail: Bender_M1@ukw.de

Supplemental Table 1: Blood parameters from control and Strumpellin-deficient mice measured on a hematology analyzer (HEMAVET). Values are mean ± s.d. (n=3).

Parameter	*Str*$^{+/+}$	*Str*$^{-/-}$	Sign.
Platelet Count [10^3/µl]	701 ± 40	702 ± 72	n.s.
Mean Platelet Volume [fl]	4.9 ± 0.10	4.9 ± 0.15	n.s.
White Blood Cells [10^3/µl]	5.8 ± 1.4	7.9 ± 1.3	n.s.
Neutrophils [10^3/µl]	0.71 ± 0.07	0.93 ± 0.25	n.s.
Lymphocytes [10^3/µl]	4.8 ± 1.27	6.6 ± 1.3	n.s.
Monocytes [10^3/µl]	0.34 ± 0.06	0.37 ± 0.14	n.s.
Red Blood Cells [10^6/µl]	10.6 ± 0.48	10.4 ± 0.18	n.s.
Hemoglobin [11.9-15.1 g/dl]	14.3 ± 0.40	14.0 ± 0.42	n.s.
Hematocrit [%]	42.9 ± 1.25	42.5 ± 1.28	n.s.
Mean Corpuscular Volume [fl]	40.3 ± 1.18	40.7 ± 0.58	n.s.
Mean Corpuscular Hemoglobin [pg]	13.4 ± 0.23	13.4 ± 0.21	n.s.
Mean Corpuscular Hemoglobin Concentration [g/dl]	33.4 ± 0.80	33.0 ± 0.10	n.s.
Red Blood Cell Distribution Width [%]	17.43 ± 0.06	17.47 ± 0.46	n.s.

Supplemental Table 2: Antibodies used in this study.

Antigen	Host	Antibody	Conjugate	Application	Company/Ref
αIIbβ3	rat	JON1	FITC	Flow cytometry	[1]
αIIbβ3	rat	JON2	FITC	Flow cytometry	[1]
αIIbβ3	rat	JON3	FITC	Flow cytometry	[1]
αIIbβ3	rat	JON6	FITC	Flow cytometry	Unpublished
αIIbβ3	rat	MWReg30	FITC	Flow cytometry	Emfret Analytics
αIIbβ3^{active}	rat	JON/A	PE	Flow cytometry	Emfret Analytics
αIIb (cytoplasmatic tail)	rabbit	AB1967	-	Immunomagnetic sorting/Western blot	Merck
αIIb	rabbit	-	-	Western Blot	ThermoFisher
β3	rat	EDL1	FITC, HRP	Flow cytometry, Western blot	[1]
α2	rat	LEN1	FITC	Flow cytometry	[2]
β1	armenian hamster	HMb1-1	FITC	Flow cytometry	BioLegend
P-selectin	rat	Wug.E9	FITC	Flow cytometry	Emfret Analytics
Strumpellin	rabbit	-	-	Western blot	Abcam
GAPDH	rabbit	-	-	Western blot	Sigma-Aldrich
Filamin A	rabbit	-	-	Western blot	Cell Signaling Technology
Fibrinogen	rabbit	-	-	Western blot	DAKO
α-tubulin	mouse	DM1A	-	Western blot	Santa Cruz
FAM21	rabbit	-	-	Western blot	Invitrogen
WASH	rabbit	-	-	IF	Invitrogen
WASH	rabbit	-	-	Western Blot	Atlas Bioscience
SWIP	rabbit	-	-	IF	ThermoFisher
CCDC53	rabbit	-	-	WB, IF	St. John´s Laboratory
anti-rabbit	goat	-	HRP	Western blot	Immuno JacksonResearch
anti-mouse	donkey	-	HRP	Western blot	Immuno JacksonResearch
CD16/CD32	rat	2.4G2	-	Blocking	Cell Signaling Technology
vWF	rabbit	-	-	IF	Dako
EEA1	rabbit	C45B10	-	IF	Cell signaling
Lamp1	rabbit	1D4B	-	IF	Abcam
anti-rabbit	goat	-	Alexa488	IF	Immuno JacksonResearch
Rab7			-	IF	Cell Signaling Technology
Rab11			-	IF	Cell Signaling Technology
GPIX	rat	Xia.B4	-	IF	Emfret Analytics

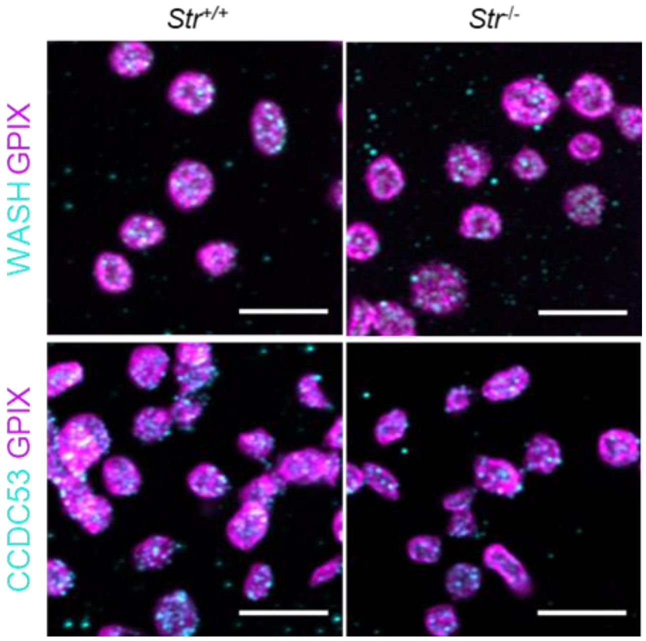

Supplemental Figure 1: WASH / CCDC53 localization and expression in control and Strumpellin-deficient megakaryocytes and platelets. Washed platelets from control and Strumpellin-deficient mice were fixed, permeabilized and probed for WASH (cyan), CCDC53 (cyan) and GPIX (magenta). Scale bar represents 5 µm.

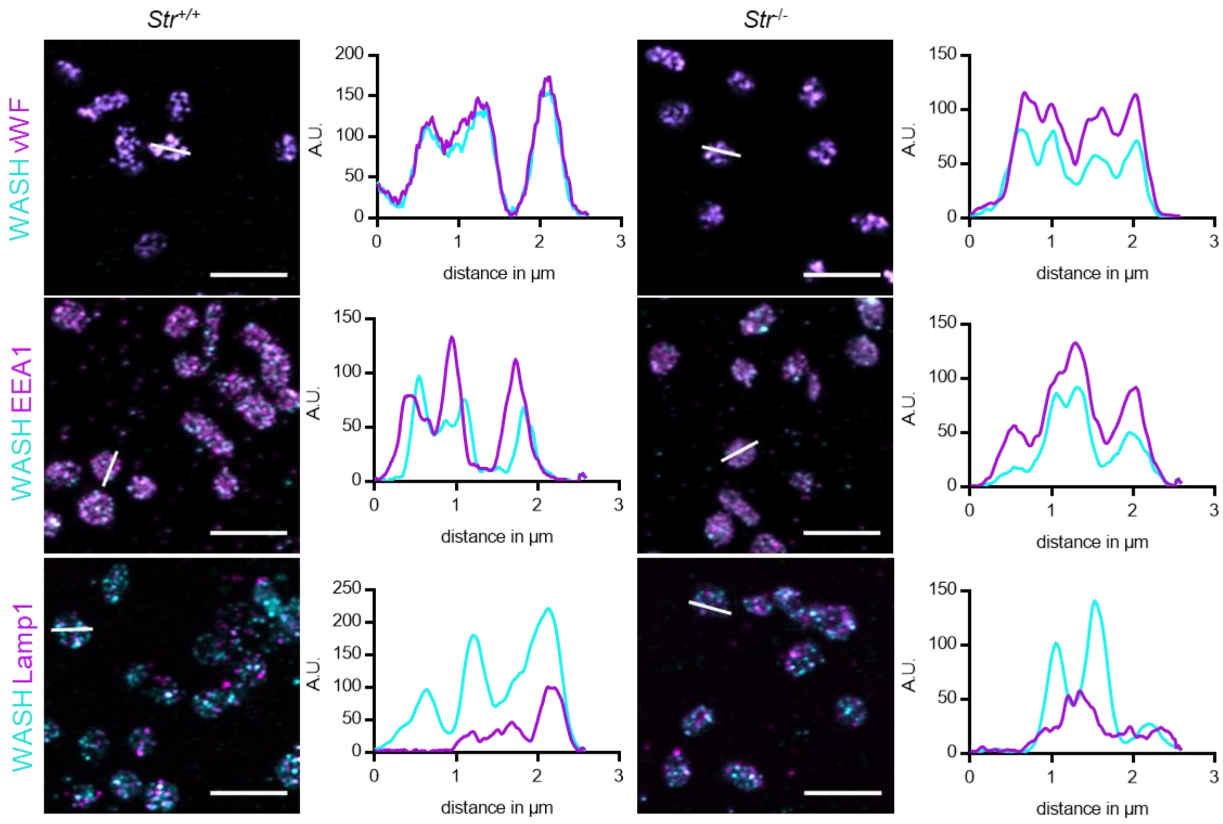

Supplemental Figure 2: Localization of WASH with vWF, EEA1 and Lamp1. Washed platelets from control and Strumpellin-deficient mice were fixed, permeabilized and probed for WASH and vWF (upper), WASH and EEA1 (middle), as well as WASH and Lamp1 (lower). White lines in the images mark the location for the line profile analysis of the respective signals (shown on the right of every image). Scale bar indicates 5 µm.

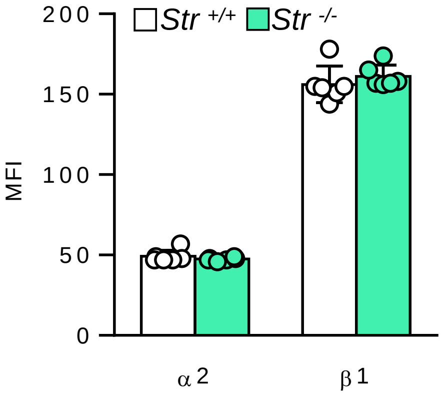

Supplemental Figure 3: Unaltered expression of integrin subunit α2 and β1 on Strumpellin-deficient platelets. Determination of total α2 and β1 surface expression with specific antibodies under resting conditions via flow cytometry. Values are mean ± s.d. (n=6).

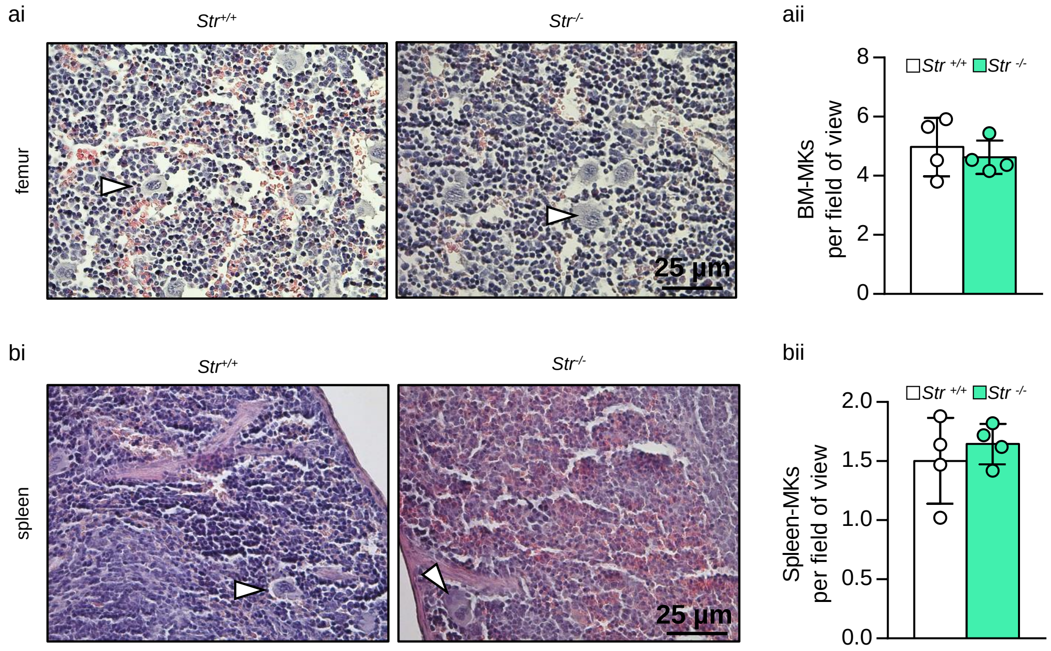

Supplemental Figure 4: Normal megakaryocyte number in the spleen and the bone marrow of Strumpellin-deficient mice. (ai, bi) Representative images of hematoxylin eosin-stained paraffin-embedded histological section of the femur **(ai)** and spleen **(bi)** from control and Strumpellin-deficient mice. White arrowhead indicates one MK in the image. Scale bar represents 25 µm. **(aii and bii)** Number of MKs in the bone marrow **(aii)** and spleen **(bii)** per field of view (64680 µm^2) based on hematoxylin eosin-stained paraffin-embedded histological sections. Values are mean ± s.d. (n=4). The experiment was performed once.

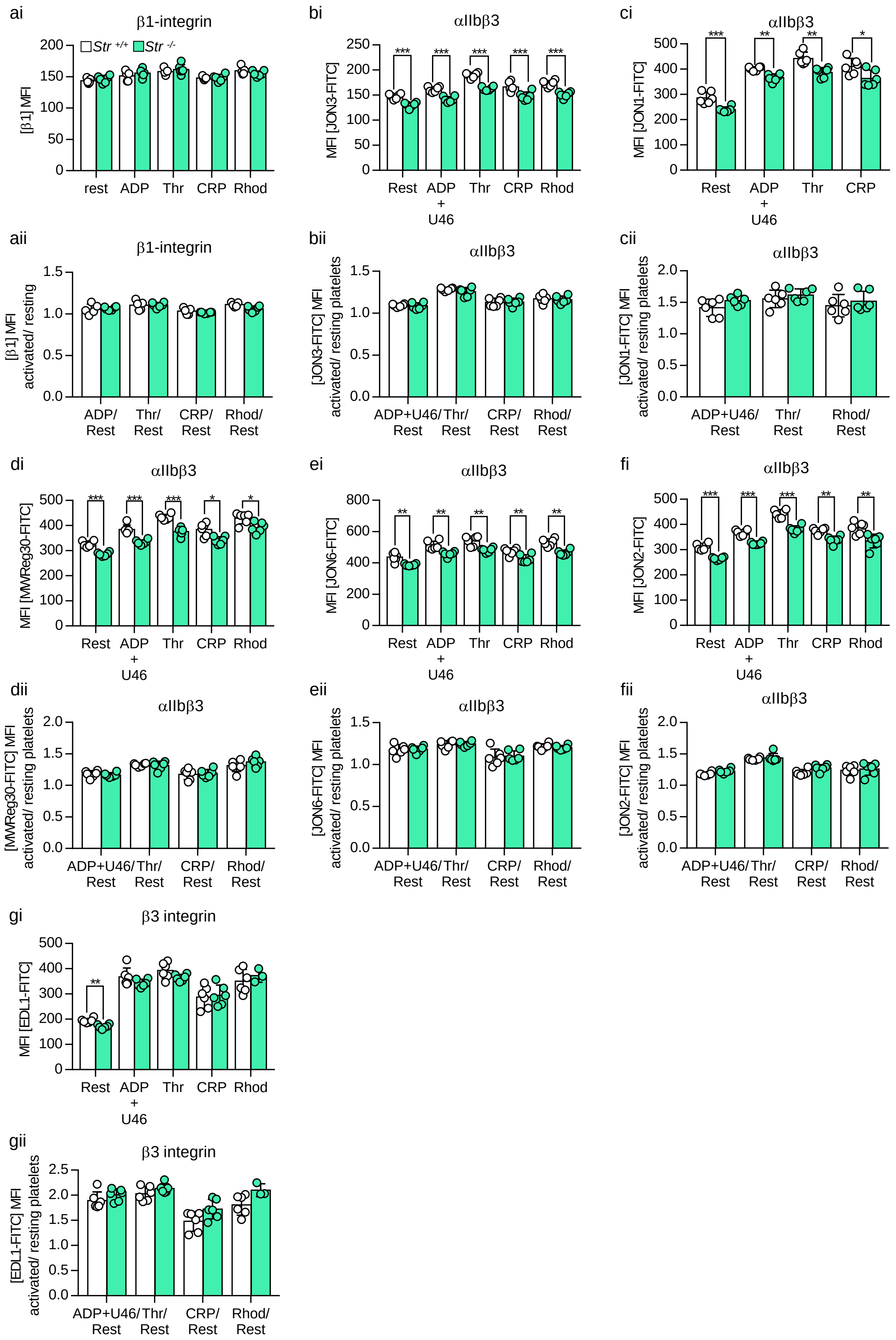

Supplemental Figure 5: Decreased surface expression of αIIbβ3 but normal expression of β1 on Strumpellin-deficient platelets under resting conditions and after activation. (ai, bi, ci, di, ei, fi, gi) Determination of total β1 (ai), αIIbβ3 (bi, ci, di, ei, fi) and β3 (gi) surface expression with specific antibodies under resting conditions and upon activation with adenosine diphosphate (ADP), U46619

(U46, thromboxane analogue), thrombin (Thr), collagen related peptide (CRP), rhodocytin (Rhod). Values are mean ± s.d. (n=6; *P<0.05; **P<0.01; ***P<0.001). (bi, ci, di, ei, fi) Different antibodies recognizing different epitopes on αIIbβ3 were used to verify the specific reduction of αIIbβ3 on the surface of platelets. **(aii, bii, cii, dii, eii, fii, gii)** Ratio of total β1, αIIbβ3 and β3 surface expression of activated versus resting platelets (ai, bi, ci, di, ei, fi, gi). Values are mean± s.d. (n=6).

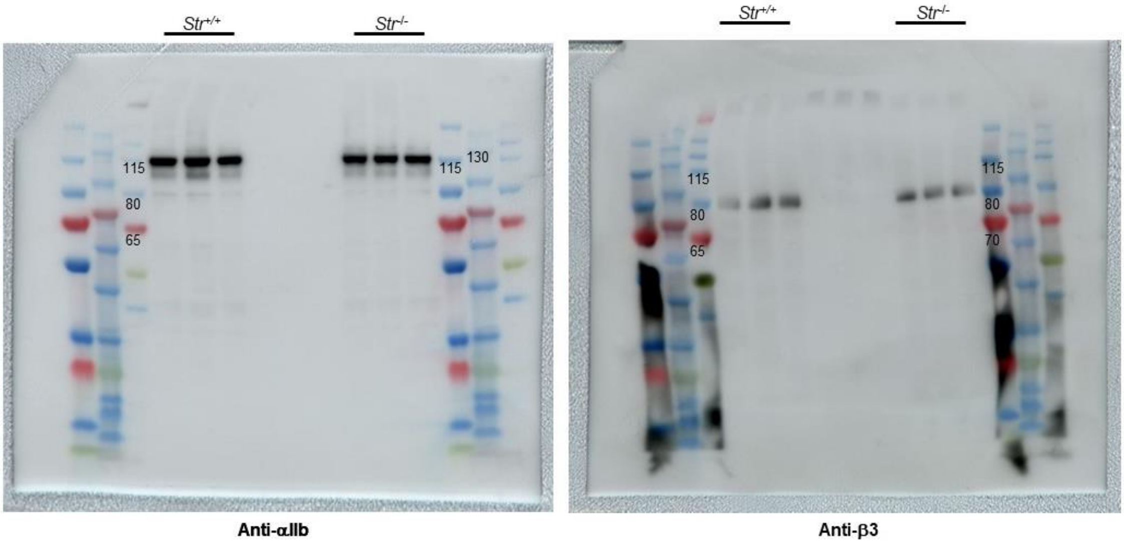

Supplemental Figure 6: αIIb and β3 subunits are expressed as full-length protein in Strumpellin-deficient platelets. Immunoblot analysis of αIIb and β3-integrin in platelet lysates from control (*Str*$^{+/+}$) and Strumpellin-deficient (*Str*$^{-/-}$) mice (n=4). Marker size indicated in kilodalton.

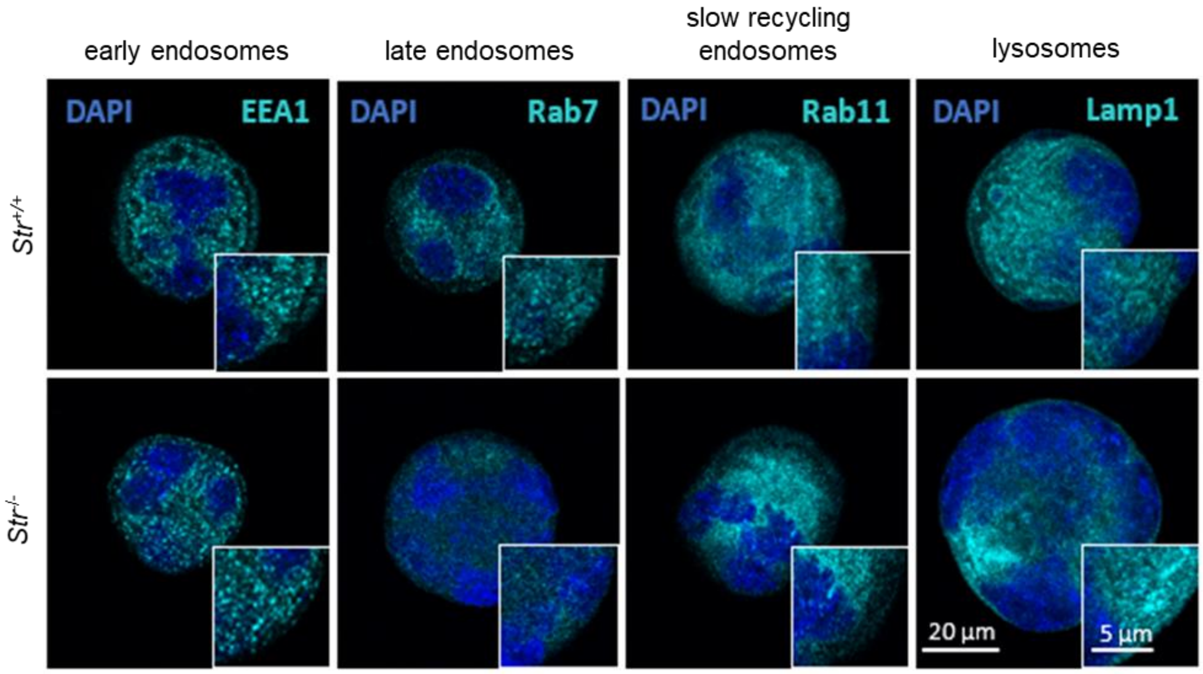

Supplemental Figure 7: Localization and expression of EEA1, Rab7, Rab11 and Lamp1 in control and Strumpellin-deficient megakaryocytes. Cultured MKs were fixed, permeabilized and stained for early endosome marker (EEA1), late endosomes marker (Rab7), slow recycling endosomes marker (Rab11) and lysosomes marker (Lamp1). DAPI was used to visualize the nucleus. Scale bars represent 20 µm (overview) and 5 µm (zoom-in).

From figure 1a

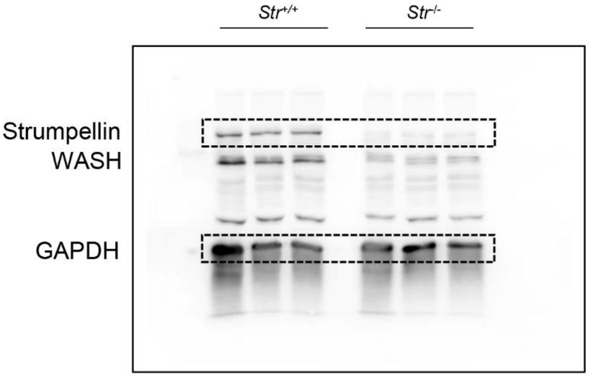

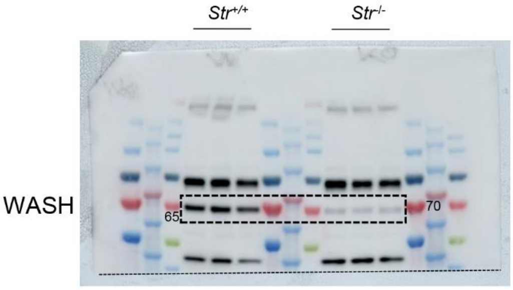

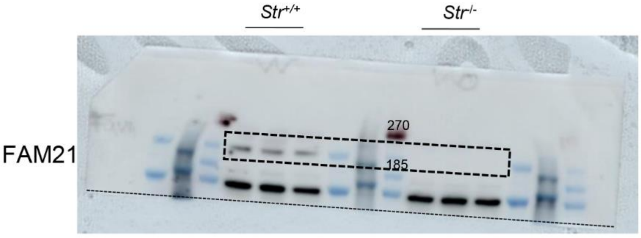

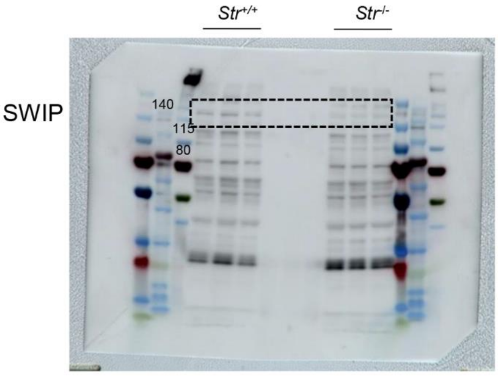

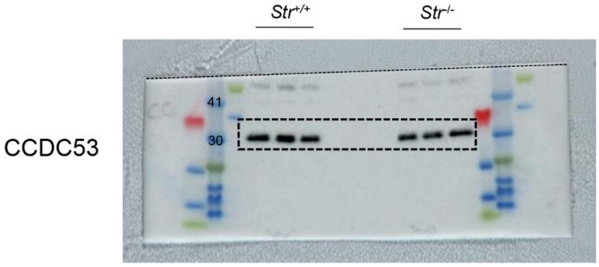

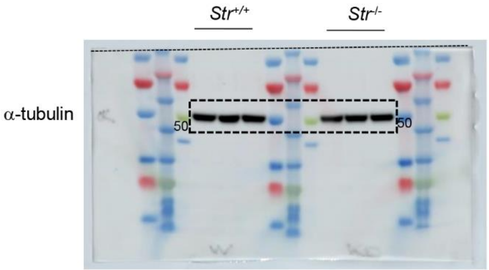

From figure 3a

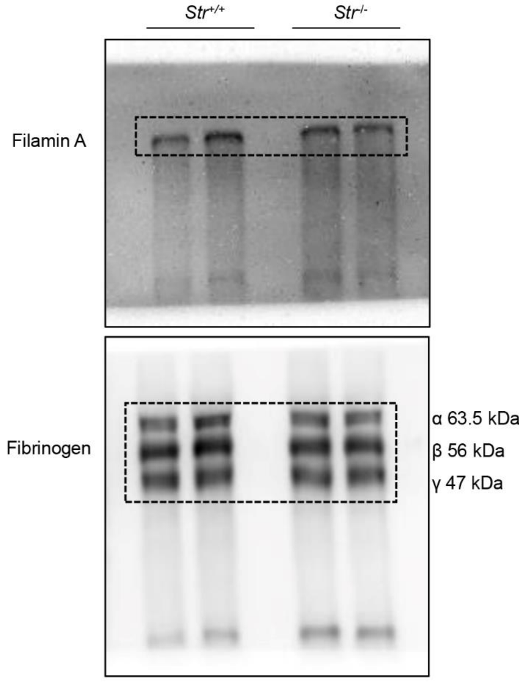

Supplemental Figure 8: Full lengths immunoblots. Black dashed lines indicate membrane was cut. Black dashed boxes indicate bands at expected protein size. Different protein ladders were used as indicated.

REFERENCES

1. Nieswandt B, Bergmeier W, Rackebrandt K, Gessner JE, Zirngibl H. Identification of critical antigen-specific mechanisms in the development of immune thrombocytopenic purpura in mice. *Blood*. 2000;96(7):2520-2527.
2. Nieswandt B, Bergmeier W, Schulte V, Rackebrandt K, Gessner JE, Zirngibl H. Expression and function of the mouse collagen receptor glycoprotein VI is strictly dependent on its association with the FcRgamma chain. *J Biol Chem*. 2000;275(31):23998-24002.
